# Microbial biotechnologies for potable water production

**DOI:** 10.1111/1751-7915.12837

**Published:** 2017-09-14

**Authors:** S. Jane Fowler, Barth F. Smets

**Affiliations:** ^1^ DTU Environment Department of Environmental Engineering Technical University of Denmark Bygningstorvet 115 2800 Kgs Lyngby Denmark

## Abstract

Sustainable Development Goal 6 requires the provision of safe drinking water to the world. We propose that increased exploitation of biological processes is fundamental to achieving this goal due to their low economic and energetic costs. Biological processes exist for the removal of most common contaminants, and biofiltration processes can establish a biologically stable product that retains high quality in distribution networks, minimizing opportunities for pathogen invasion.

The WHO suggests that humans require an absolute minimum of 7.5 L of water per day, while a minimum of about 20 L of water per person per day is recommended to ensure adequate hygienic standards. With a population of 7.5 billion, this works out to 150 billion litres of safe freshwater daily, globally. Much more than this is generally consumed in developed nations, while less than adequate amounts of safe water are available in some regions. Although sufficient freshwater resources exist to meet global water needs, the major limitation is the lack of infrastructure in some regions for production and distribution of safe water. The global provision of safe water is a key aim of UN Sustainable development goal (SDG) 6, but this goal also intersects closely with SDG 13, climate change, requiring energy efficiency and minimal GHG emissions, and SDG 12, requiring sustainable global water consumption and production patterns and reductions in pollution of water resources. Safe drinking water has a quality that would not present any significant risk to health over a lifetime of consumption. While physical and chemical disinfection processes may remain essential to reduce the pathogenic burden during water treatment, we believe that increased exploitation of microbiological processes for drinking water treatment is the most sustainable way forward for the global provision of safe water. Biological drinking water treatment processes are available for the removal of a wide range of chemical contaminants, are less costly and less energy intensive than advanced chemical or physical treatment methods and are robust over a wide range of operating conditions and water qualities. Furthermore, they reduce the use of potentially hazardous chemicals and typically result in complete mineralization of contaminants, rather than concentration in a waste stream, which then necessitates specialized treatment and/or disposal. In addition, recent and ongoing research indicates that providing biologically stable water can be accomplished by fostering the presence of a natural resident, non‐pathogenic drinking water microbiome that can resist pathogen invasion in water supplies, which can be achieved through the use of biological drinking water treatment processes.

The major sources of drinking water are surface water and groundwater. Both forms of water are generally not safe at the source and require some form of treatment to be considered potable. To ensure adequate water quality, regulatory guidelines exist for (i) biological contaminants (pathogenic bacteria, protozoa, viruses and helminths), (ii) inorganic chemicals (metals, oxyanions, nitrogen species and radionuclides), and (iii) organic chemicals (natural organic matter and synthetic organic chemicals from agricultural, industrial and residential use). In regions where disinfection is used in drinking water treatment, disinfectant residuals and disinfection by‐products are also typically regulated due to their potential adverse health effects. In addition, physical aspects of the water including colour, odour and taste also contribute to water quality.

Historically and up to the present day, microbial processes have been used in the production of potable water. Biological drinking water treatment has been widespread since the 1800s in the form of slow sand filtration or bank filtration (Schubert, 2002; Logsdon *et al*., 2011). While historically, biological water treatment was empirical, we now have the technology and tools to understand the structure and function of the microbial communities involved in biological water treatment, potentially enabling control and optimization, making these processes even more attractive. Unfortunately, the use of biological processes for drinking water production has been in decline in recent years due to misconceptions regarding a relationship between the presence and exploitation of microbes in drinking water treatment and the presence of pathogens. In North America (and many regions around the world), disinfection is routinely used in an effort to *sterilize* drinking water. Despite the use of disinfectants and the presence of disinfectant residuals in distribution systems, 10^3^–10^9^ bacteria per mL are still present in drinking water at the tap (Hammes *et al*., 2008; Lautenschlager *et al*., 2010; Nescerecka *et al*., 2014). It is virtually impossible to completely remove microbes in water while delivering a safe product to consumers, and the use of disinfectants results in selection of (potentially pathogenic) bacteria that are resistant to disinfectants in distribution systems (Chiao *et al*., 2014). Disinfection can result in the creation of unfilled ecological niches, increasing opportunities for invasion of water supplies by foreign bacteria. The concomitant production of assimilable organic carbon (AOC) from the reaction of residual disinfectant with recalcitrant organic matter also results in new niches and increased microbial carrying capacity (Reckhow *et al*., 1990; Fass *et al*., 2003). With long residence times in distribution systems, disinfectant residual is often lost and is associated with microbial regrowth, implying that disinfection merely delays microbial growth rather than prevents it (Servais *et al*., 1995). In all cases, this results in decreased biological stability of water. Combined with potential long‐term health effects associated with disinfectants and disinfection by‐products (Sadiq and Rodriguez, 2004), this leads us to propose that the use of disinfectants should be limited. Removal of pathogens preferably takes place early and is followed by biological treatment for the removal of disinfection by‐products and other chemical contaminants. In this way, a biologically stable product with low nutrient concentrations is obtained in which the absolute abundance and composition of the microbial community does not vary substantially throughout the distribution network (Prest *et al*., 2016). This drinking water microbiome can be established using biological filtration steps that result in a natural resident community in the distributed water (Pinto *et al*., 2012; Lautenschlager *et al*., 2014). Ideally, this microbial community will be diverse and therefore will be more resistant to invasion than disinfected communities where unfilled niches would exist (Kinnunen *et al*., 2016). Nonetheless, further research on factors that improve resistance to invasion, and examination of the capacity of common pathogens to invade drinking water microbiomes, needs to be conducted.

The most common use of microbial biotechnology in drinking water treatment is biological filtration. This involves the filtration of oxic or oxygenated water through granular media such as sand, granular activated carbon (GAC) or anthracite and may include slow sand filtration, rapid sand filtration (gravity or pressurized) or bank filtration. Microorganisms grow on the surface of the medium and are involved in removing a range of substances depending on the source water (Gülay *et al*., 2014). Biological filtration is used for the removal of inorganic compounds (e.g. ammonium, nitrite, sulphide, methane, iron and manganese; de Vet *et al*., 2011; Tatari *et al*., 2012, 2016; Lee *et al*., 2014), organic compounds (with or without prior ozonation) including natural organic matter (DeVries *et al*., 2012) as well as a wider array of organic pollutants such as pesticides (Hedegaard and Albrechtsen, 2014; Hedegaard *et al*., 2014), pharmaceuticals (Petrovic *et al*., 2009), disinfection by‐product precursors (McKie *et al*., 2015) and arsenic (Katsoyiannis and Zouboulis, 2004). Adaptation of biological filtration systems with zero valent iron can also be used for the removal of radionuclides such as uranium (Gottinger *et al*., 2013), and other adsorbents could be incorporated into biological filters for the removal of heavy metals such as lead and cadmium (Ali and Gupta, 2007). In some regions, biological filtration is used as the sole means of water treatment in single or successive rapid sand filters for the treatment of aerated groundwater (e.g. Denmark) or as a sequential ozonation, and filtration treatment train for surface water treatment (e.g. Zurich). In regions where oxidative treatment is used, biological filtration is commonly used after oxidation to remove the produced AOC. Bioaugmentation of biological filters is increasingly being investigated for the removal of recalcitrant contaminants (Ho *et al*., 2007; Benner *et al*., 2013; Horemans *et al*., 2017). Bioaugmentation has the potential to increase the diversity of pollutants and micropollutants that are degradable in biological filters. This is an important development, as increasing water re‐use necessitates improved technologies for pollutant removal. For bioaugmentation to be a successful strategy in the long‐term, further research is needed to understand the ability of bioaugmented organisms to invade and establish in a resident microbial community, and then grow under the oligotrophic conditions typical of drinking water filters. The current effort at elucidating the complete composition and metabolic potential of biofiltration communities (e.g. Gülay *et al*., 2016; Palomo *et al*., 2016), the availability of different granular media types, and the lower footprint of pressurized filtration units, is leading the way towards truly engineered biofiltration processes for potable water preparation.

Membrane‐based biofilm reactors have also been investigated for the removal of a wide range of recalcitrant drinking water contaminants. Reductive treatment of oxidized compounds such as bromate, nitrate, selenate/selenite, chromate, perchlorate/chlorate/chlorite and arsenite – which can be primary pollutants in various groundwater sources – can be achieved using membrane biofilm reactors (MbfR) fed with hydrogen or methane as electron donor (Nerenberg and Rittmann, 2004; Rittman, 2006). In these cases, the pollutant to be removed is respired by the microbial community, which grows on the membrane surface, and electron donor is supplied via the membrane lumen. MbfRs have also been tested for the treatment of diverse organic contaminants including BTEX and chlorinated solvents (Syron and Casey, 2008) and pharmaceuticals (Kim *et al*., 2010). While the MbfR allows for very small footprint reactors, where exact dosing control of electron donors can lead to cost‐efficient biotreatment technologies with engineered biofilms (Nerenberg, 2016), it remains to be examined whether the increased operational complexity (supply of gasses) and investment costs (membranes) provides a sufficient advantage over traditional biofiltration solutions that would depend on soluble electron donor additions (e.g. Upadhyaya *et al*., 2010).

In summary, biological methods for the removal of common as well as context‐specific and recalcitrant contaminants are available. We believe that increased exploitation of biological processes for drinking water treatment is the best way to achieve the provision of safe water globally. Biological processes are generally less costly and less energy intensive than advanced chemical and physical removal processes and can be effective for the removal of the majority of relevant contaminants. Furthermore, biological filtration processes at the end of treatment trains ensure the presence of a biodiverse drinking water microbiome that may be more effective than disinfection at reducing pathogen invasion.

## Conflict of interest

None declared.
